# New nanoformulation of rapamycin Rapatar extends lifespan in homozygous *p53^−/−^* mice by delaying carcinogenesis

**DOI:** 10.18632/aging.100496

**Published:** 2012-10-29

**Authors:** Maria Comas, Ilia Toshkov, Karen K. Kuropatwinski, Olga B. Chernova, Alexander Polinsky, Mikhail V. Blagosklonny, Andrei V. Gudkov, Marina P. Antoch

**Affiliations:** ^1^ Departments of Molecular and Cellular Biology, Roswell Park Cancer Institute, Buffalo, NY, USA; ^2^ Cleveland Biolabs, Buffalo, NY 14203, USA; ^3^ Tartis Aging, Inc, Buffalo, NY14203, USA; ^4^ Cell Stress Biology, Roswell Park Cancer Institute, Buffalo, NY USA; ^5^ Cancer Research Program, Garvan Institute of Medical Research, Darlinghurst, New South Wales, Australia

**Keywords:** mTOR pathway, p53−/−, lifespan, rapamycin, oral formulation, cancer prevention

## Abstract

The nutrient-sensing mTOR (mammalian Target of Rapamycin) pathway regulates cellular metabolism, growth functions, and proliferation and is involved in age-related diseases including cancer, type 2 diabetes, neurodegeneration and cardiovascular disease. The inhibition of mTOR by rapamycin, or calorie restriction, has been shown to extend lifespan and delays tumorigenesis in several experimental models suggesting that rapamycin may be used for cancer prevention. This requires continuous long-term treatment making oral formulations the preferred choice of administration route. However, rapamycin by itself has very poor water solubility and low absorption rate. Here we describe pharmacokinetic and biological properties of novel nanoformulated micelles of rapamycin, Rapatar. Micelles of Rapatar were rationally designed to increase water solubility of rapamycin to facilitate oral administration and to enhance its absorption. As a result, bioavailability of Rapatar was significantly increased (up to 12%) compared to unformulated rapamycin, which concentration in the blood following oral administration remained below level of detection. We also demonstrated that the new formulation does not induce toxicity during lifetime administration. Most importantly, Rapatar extended the mean lifespan by 30% and delayed tumor development in highly tumor-prone *p53^−/−^* mice. Our data demonstrate that water soluble Rapatar micelles represent safe, convenient and efficient form of rapamycin suitable for a long-term treatment and that Rapatar may be considered for tumor prevention.

## INTRODUCTION

Rapamycin (or Sirolimus) is a macrolide antibiotic that was first isolated from *Streptomyces hydroscopicus* and was initially utilized as an antifungal agent [1,2]. Under the name of Rapamune, it is now used as an immunosuppressant to prevent organ rejection after transplantation. Rapamycin inhibits the nutrient-sensing mTOR (mammalian Target of Rapamycin), a conserved protein kinase that controls cellular growth and metabolism. The mTOR signaling pathway is activated by nutrients, growth factors, hormones, cytokines, and cellular energy status. When nutrients and growth factors are abundant, mTOR promotes protein synthesis, ribosome biogenesis, angiogenesis, cell cycle progression and cytoskeleton re-organization (reviewed in [3]-5]).

Recent data demonstrated that rapamycin extends life span in various model organisms including mammals [4-6]. The life-long administration of rapamycin inhibits age-related weight gain, decreases aging rate and increases lifespan of inbred [7] and genetically heterogeneous [6] mice. Previous data has demonstrated that rapamycin significantly delayed the onset of spontaneous carcinogenesis both in normal (129/Sv [7]) and cancer-prone (HER-2/neu transgenic [8] and *p53^+/−^*[9]) mice. Importantly, the anti-cancer effect of rapamycin in *p53^+/−^* mice was blunted when treatment started at the age of 5 months [9] suggesting that rapamycin does not directly inhibit tumor growth but rather has an indirect effect.

Since rapamycin exhibits poor water solubility and instability in aqueous solutions, its clinical use through oral administration requires development of special drug design such as complex nanoparticle formulation to facilitate increased bioavailability and efficacy. Therefore, various oral formulations, such as inclusion complexes [10,11], liposomes [12], nanocrystals [13], and solid dispersion [14] have been developed and tested in pre-clinical and clinical studies. In this study, we tested the biological activity of a novel formulation of rapamycin, Rapatar. This formulation is based on Pluronic block copolymers as nanocarriers, which serves to improve water solubility of the drug, and to enhance various biological responses favorable for therapeutics, such as activity of drug efflux transporters (reviewed in [15]). We show that Rapatar has significantly higher bioavailability after oral administration when compared to unformulated rapamycin. We also show that Rapatar effectively blocks mTOR in mouse tissues. Moreover, life-long administration of Rapatar increases lifespan and delays carcinogenesis in highly tumor-prone *p53^−/−^* mice.

## RESULTS

### Rapatar is efficiently absorbed and systemically distributed and effectively inhibits mTOR *in vivo*

**Figure 1:**
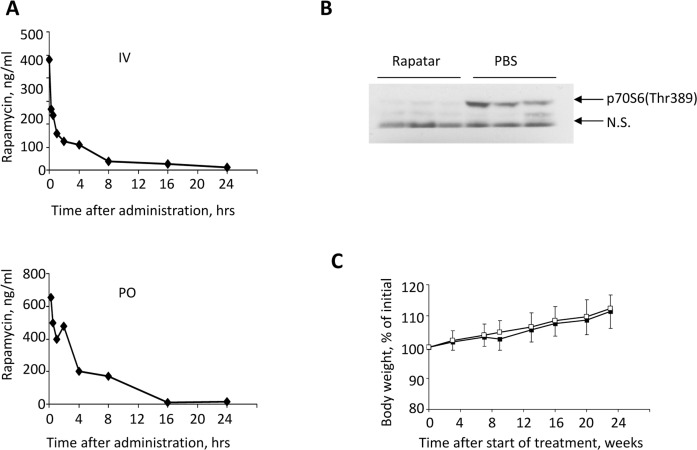
Pharmacokinetic and biological characteristics of Rapatar. (**A**) Rapamycin concentration-time profile in blood after intravenous (IV, top) and oral (PO, bottom) administration of Rapatar to mice (mean values, n = 3). A single dose of Rapatar was administered either IV (0.4mg/kg) or PO (4mg/kg). Blood samples were collected at designated times and analyzed for rapamycin by **LC/MS/MS**. (**B**) Rapatar blocks mTOR activation in vivo. Six C57/Bl/6J mice were food-deprived for 18 hrs. At the end of fasting period animals received either Rapatar (0.5mg/kg) or PBS via gavage and were allowed access to food. One hour later animals were sacrificed, livers were dissected and protein lysates were analyzed for mTOR activity by probing with p70S6(Thr389) antibody. (**C**) No acute or long-term toxicity are associated with PO administration of Rapatar. C57Bl/6J male mice received either Rapatar or PBS starting 8 weeks of age (10 mice/group) for 24 weeks according to the protocol described above. No loss in body weight was detected in experimental group throughout the treatment period. Both experimental and control groups showed similar gain in body weight with age.

To compare the absolute and relative bioavailability and other pharmacokinetic properties of Rapatar with those of an unformulated rapamycin, we administered both compounds as a single dose to female ICR mice. Rapatar was administered intravenously (IV) or orally (PO) at a dose of 0.4 mg/kg and 4 mg/kg respectively, while rapamycin was administered PO at 4 mg/kg. Blood samples were collected at different times after administration and analyzed for rapamycin by mass spectrometry LC/MS/MS). Pharmacokinetic values of the area under the curve (AUC), the maximum drug concentration (C_max_), the time of peak concentration (T_max_), and the absolute bioavailability (F) were calculated from whole blood drug concentration-time data (Fig. 1A). Importantly, following oral administ-ration, rapamycin could only be detected in whole blood samples of mice that received Rapatar whereas its concentration in blood of rapamycin-treated mice was beyond the level of detection. As shown in Table 1, when compared to unformulated rapamycin, Rapatar demonstrated very fast absorption (T_max_ 15 min) and significant increase in AUC value with mean T_1/2_ extending to 6.4 hours. Consequently, a single oral administration of Rapatar resulted in 12% bioavailability, which is comparable with commercially available formulations used in clinical practice (14% when administered orally in combination with cyclosporine A).

**Table 1:** Pharmacokinetic parameters of unformulated rapamycin and Rapatar in C57Bl/6J mice. Abbreviations: C_max_ – the peak concentration; T_max_ – time taken to reach peak concentration; AUC – area under the curve; F – absolute bioavailability

	Units	Rapamycin, IV 0.4mg/kg	Rapatar, PO 4mg/kg
Dose amount	ng	10.4	104
Dosage	ng/kg	400	4000
Cmax	ng/ml	958	656
Tmax	hr	0.04	0.25
AUC	ng-hr/ml	2634.6	3161.5
Half-life	hr	6.4	N/A
F	%	100	12

Ribosomal protein S6 is a substrate of mTOR, and therefore phospho-ribosomal protein S6 is a marker of mTOR activity [16-19]. To test whether Rapatar inhibits mTOR activity in vivo, we compared levels of phosphorylated S6 (pS6) in livers of wild type C57Bl/6J mice, in which mTOR was suppressed by a period of food deprivation. Rapatar (0.5mg/kg or PBS were given by gavage at a time when animals were allowed access to food. Fig. 1B shows that S6 is highly phosphorylated in livers of control animals indicating mTOR activation in response to food. In contrast, in animals that received Rapatar, S6 phosphorylation was reduced ~10-fold. Thus, Rapatar successfully inhibits mTOR activity in the liver in vivo.

To test whether life-long administration of Rapatar causes in vivo toxicity, we administered it to wild type C57Bl/6J mice at 0.5 mg/kg via gavage according to protocol described in Materials and Methods section.

Rapatar- and PBS-treated animals were monitored for any signs of toxicity by visual inspection and body weight measurements. Mice receiving Rapatar maintained a healthy appearance with physical activities and body weights comparable to the control mice (Fig. 1C).

### Rapatar increases lifespan of *p53^−/−^* mice

**Figure 2:**
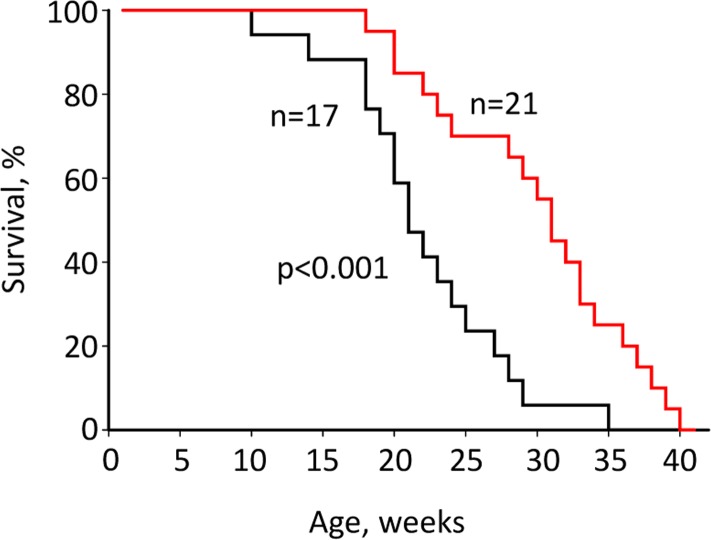
Rapatar increases lifespan in *p53^−/−^* mice. Mice received Rapatar at 0.5 mg/kg via gavage according to the schedule described in Materials and Methods. Rapatar increased lifespan from 23 to 31 weeks (p<0.001, Mantel-Cox log-rank test).

Our data showed that Rapatar effectively inhibits mTOR *in vivo*. Suppression of mTOR by rapamycin has been shown to increase lifespan in various model organisms including mice [6-8,20-25]. To test whether Rapatar can extend lifespan, we administered it to mice with targeted disruption of tumor suppressor p53. *p53^−/−^* mice are characterized by increased carcinogenesis and reduced lifespan (reviewed in [26]. Twenty p53^−/−^ mice received Rapatar starting 8 weeks of age at a dose of 0.5mg/kg according to the schedule described in Material and Methods. Another group of 17 *p53^−/−^* mice received PBS as control. Throughout the experiment, animals were monitored for tumor development by visual inspection and total body weight measurements. Both Rapatar- and PBS-treated *p53^−/−^* mice die early in life due to a high rate of spontaneous carcinogenesis, which is characteristic for this mouse model. However, treatment with Rapatar resulted in an overall significant increase in median survival of *p53^−/−^* mice from 23 (±10) weeks in the control group to 31 (±1.5) weeks in the experimental group (Fig. 2A).

To gain insight into the potential mechanism of increase in survival of Rapatar-treated animals, we performed a detailed histological analysis of all tissues collected from each individual animal in the course of the experiment (summarized in Table 2). Based on this analysis, 82% of mice in the control group (14 out of 17) developed lymphomas whereas 12% (2 out of 17) developed sarcomas. One animal showed the presence of both sarcoma and lymphoma and one animal developed myeloid leukemia. This spectrum of tumors is characteristic to *p53^−/−^* mice and comparable to previous reports [27]. The mice developed these spontaneous neoplasms from 2 to over 8 months of age with an average latency time of 161 days. When compared to the control group, Rapatar-treated mice showed later appearance and delayed progression of spontaneous tumors. They arose from 4.5 to over 9.5 months, with average latency of 261 days; one animal remained tumor-free until the end of the experiment. Interestingly, the incidence of sarcomas in Rapatar-treated mice was increased to 30% compared to 17% in control group (Table 2); however the number of animals used in the experiment was not enough to obtain a statistically significant difference.

**Table 2:** Summary of histological analysis. Tissues of 17 control and 20 Rapatar-treated *p53^−/−^* mice were evaluated for the presence of tumor cells. The type of tumors and the stage of their development were determined as described in Materials and Methods. The incidence of sarcomas in Rapatar-treated *p53^−/−^* mice was higher than in control group (30% and 17% respectively); however, due to a relatively small group size, statistical significance was not achieved (p=0.2; Fisher's exact test)

	Initial Lymphoma	Advanced Lymphoma	Sarcoma	Leukemia	Tumor-free
Rapatar	7 (35%)	6 (30%)	6 (30%)	1 (5%)	1 (5%)
PBS	4 (23%)	10 (58%)	3 (17%)	1 (6%)	0

**Figure 3:**
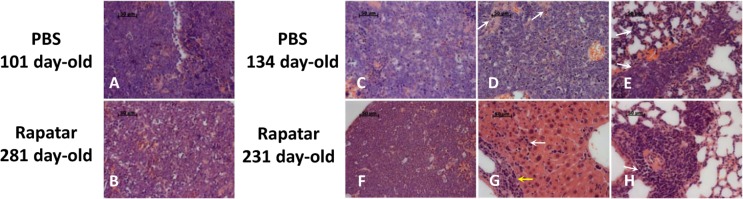
Rapatar delays development of lymphomas in *p53^−/−^* mice. (**A)** Representative initial lymphoma developed in control mouse at the age of 101 days. (**B**) Similar appearance of lymphoma in Rapatar-treated mouse at 281 days of age. Both **A** and **B** show monotonous infiltrate of medium-sized neoplastic cells with round nuclei, fine chromatin, indistinct nucleoli, and numerous mitotic figures and apoptotic cells. (**C**) Advanced lymphoma in 134-day old control mouse with metastases in liver (**D**) and lung (**E**). (**D**) Metastasis in liver showing the extensive spread of neoplastic cells effaces the normal structure and only minimal remnants of hepatocytes (marked by arrows). (**E**) Metastasis in the lung showing neoplastic infiltrates in perivascular area and in the alveolar walls (arrows) (**F**) Advanced lymphoma with pathological changes similar to shown in C in the thymus of 241day-old Rapatar-treated animal with metastasis in liver (**G**) and lung (**H**). (**G**) Metastasis in liver showing neoplastic infiltrates in portal tract (yellow arrow) and sinusoids (white arrow). (**H**) Metastasis in the lung showing perivascular neoplastic infiltrate (arrow).

**Figure 4:**
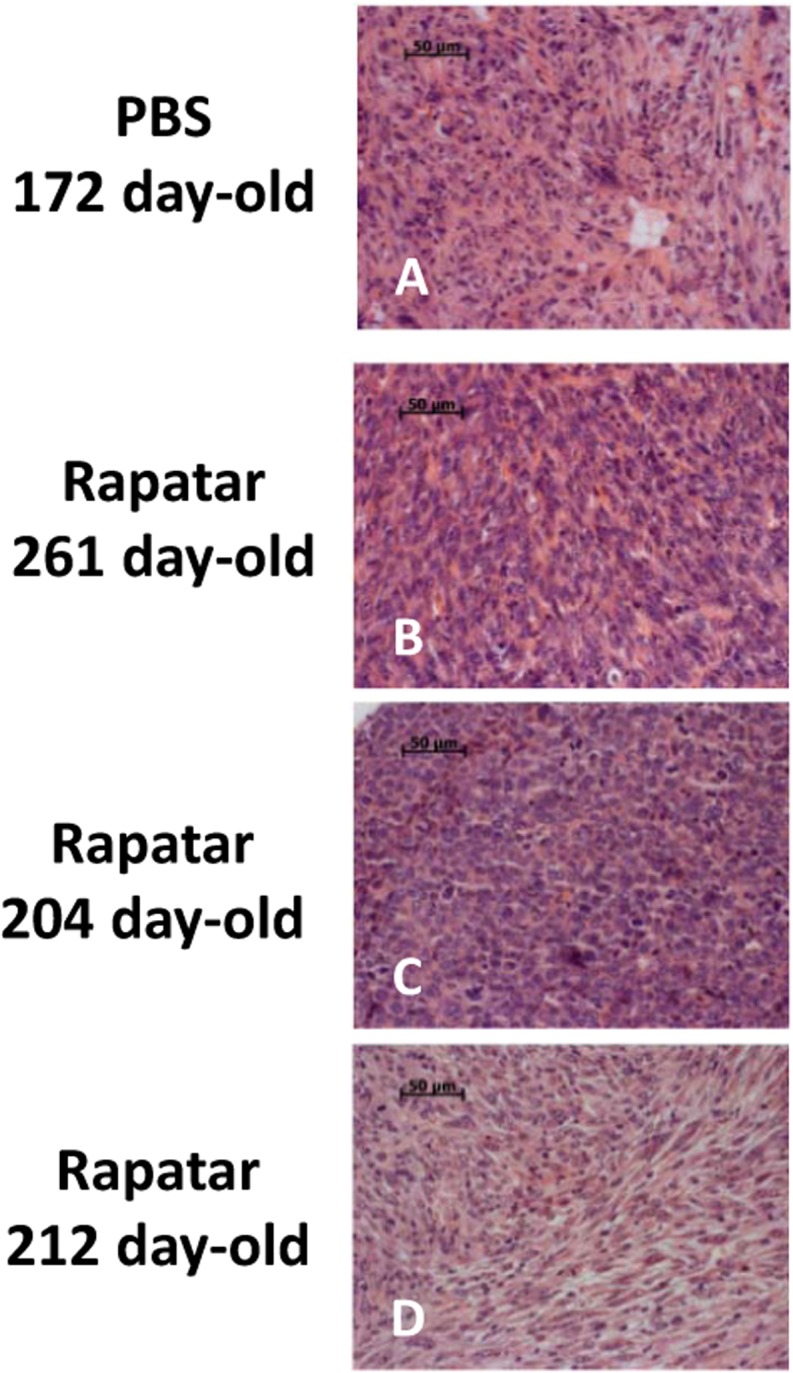
Rapatar delays development of sarcomas in *p53^−/−^* mice. (**A**) Liver sarcoma in 172-old control mouse. (**B,C**) Sarcoma developed in 261 day- and 204 day-old Rapatar-treated mice. No metastases are detected. D. Sarcoma in 212-day old Rapatar-treated mouse with metastases in the lung.

Since lymphomas represented the major type of tumor in both groups, we performed a detailed pathological evaluation of individual tumors. Based on the severity of pathological changes, the developmental stage, and involvement of non-lymphoid tissues, all lymphomas were graded as initial or advanced. Initial lymphomas mainly involved thymus and were presented macroscopically as enlarged masses. Under the microscope they were seen to be composed of broad sheets of densely packed rather uniform large lymphoblastic cells, with little or sparse cytoplasm that completely obliterated the normal thymus structure and cortical and medullary zones. In most cases, neoplastic lymphoid cells expanded through the thymic capsule and spread through the mediastinal fat, lymph nodes, along peritracheal and periaortal spaces, even infiltrating lungs and pericardium with limited penetration of the myocardium. Such lymphomas with predominantly local involvement were designated provisionally as initial. Tumors were graded as advanced when the rise of the malignancy and aggressiveness of the lymphoma cells resulted in metastases and infiltration into spleen, liver, lung, kidney, mesentery lymph nodes, testis, and bone marrow. Based on this designation, the proportion of the initial lymphomas in Rapatar-treated group was larger compared to controls (35% and 23% for experimental and control groups respectively) suggesting that Rapatar slows down tumorigenesis. Consistently, the proportion of the advanced disseminated lymphomas, spreading to other organs in Rapatar-treated group was smaller than in control (30% and 58% respectively). Although histopathological appearance of lymphomas and sarcomas were very similar in control and experimental groups, Rapatar-treated mice develop tumors significantly later in life (Fig. 3 and 4). Based on these data we concluded that Rapatar increased lifespan of *p53^−/−^* mice by delaying tumorigenesis.

## DISCUSSION

The mTOR signaling pathway is a key coordinator of cell growth and cell proliferation in response to a variety of environmental conditions. Its deregulation has been implicated in many pathological conditions, including those that are associated with aging, such as cancer, type 2 diabetes, neurological and cardiovascular disorders (reviewed in [28,29]). Furthermore, the activation of the mTOR pathway is the most universal alteration in cancer [30]. Several analogs of rapamycin (rapalogs) have been approved for cancer therapy [31-35] and numerous clinical trials are underway. However, as anti-cancer drugs rapamycin and other rapalogs showed modest efficacy. There are several reasons that can explain relatively low therapeutic effect. First, rapamycin itself is not cytotoxic. Additionally, mTOR inhibition activates several feedback loops that drive mitogenic signaling (reviewed in [28,36]). Therefore, it is still not quite clear whether rapamycin exhibits direct antitumor activity or whether it acts in a more indirect systemic way. Our previous data [9] and data presented here show that rapamycin delays carcinogenesis in tumor-prone *p53^+/−^* and *p53^−/−^* mice, most likely by slowing down the process of aging. If this is the case, than rapamycin can be considered as a tumor-preventive agent (i.e. administration is required before tumor initiation). This necessitates the development of efficacious nontoxic rapamycin-formulations that could be taken orally for extended periods of time. Here we show that oral administration of Rapatar results in high systemic bioavailability and does not induce toxicity during life-long administration. Importantly, biological effects of Rapatar were prominent at low doses (0.5 mg/kg) and intermittent schedules. Taken together, our data suggest that Rapatar is a promising candidate for clinical use as an effective cancer prevention drug.

## MATERIALS AND METHODS

### Materials

Rapamycin was purchased from LC Laboratories (Woburn, MA). Polymeric formulation of rapamycin (Rapatar) was developed by Tartis Aging, Inc. using Pluronic block co-polymers [15] according to the following protocol. One gram of rapamycin was dissolved in 25 ml of ethanol. The resulting solution was mixed with 5 grams of Pluronic L-92 (BASF) and 2 grams of citric acid dissolved in 200 ml of 20% Pluronic F-127 (BASF) solution in ethanol and water mixture (97:3 v:v). The solution was then incubated at 20–25°C for 30 minutes with constant stirring. The ethanol was removed using Speedvac and the formulation was further dried using high vacuum.

### Animals

ICR female mice were obtained from Charles River. C57Bl/6J mice were obtained from Jackson Laboratory. p53−/− mice on C57Bl/6J background originally obtained from Jackson Laboratory, were housed and bred at the Department of Experimental Animal Resources of Roswell Park Cancer Institute. For pharmacokinetic analysis, three groups of 8 weeks old ICR female mice received a single dose of either Rapatar (2 groups) or rapamycin. Rapatar was administered via gavage at 4mg/kg in 0.5% methyl cellulose or IV at 0.4mg/kg in PBS. Rapamycin was administered via gavage at 4mg/kg in 0.5% methyl cellulose.

For estimating potential long-term toxic effects of Rapatar, two groups of C57BL/6J mice received the drug at a dose of 0.5 mg/kg via gavage once a day for 5 consecutive days, followed by 9-day interval without treatment. Mice were maintained on this treatment schedule for 24 weeks and were weighed weekly. Control mice receive PBS according to the same schedule.

For longevity studies, 38 *p53^−/−^* male mice were randomly divided into two groups. Twenty one experimental animals received 0.5 mg/kg Rapatar and 17 animals received PBS according to the above described schedule. Treatment started at 8 weeks of age and continued until tumor appearance was visually observed or dramatic loss of weight, indicative of tumor appearance, was detected. At this point, mice were sacrificed and examined for gross pathological changes. Tumors, heart, kidney, liver, lungs, thymus and spleen were collected for histological evaluation. All procedures were approved by the Institutional Animal Care and Use Committee of Roswell Park Cancer Institute.

### Pharmacokinetic study

Whole blood was collected into EDTA-blood tubes 0.5, 1, 2, 4, 8, 16 and 24 hours after administration of either Rapatar or unformulated rapamycin. Tubes were inverted a few times, and stored on ice in dark container during the experiment. At the end of the experiment, all samples were placed for storage at −70°C in a light-protected container. Frozen blood samples were submitted to the Rocky Mountain Instrumental Laboratory (Fort Collins, Co) for LC/MS/MS analysis of rapamycin. Pharmacokinetic analysis was performed using data from individual mice for which the mean and standard error of the mean (SEM) were calculated for each group using PK Solutions software (Version 2.0).

### Western blot analysis

In order to maximize and be able to detect p70S6 phosphorylation [37,38], six C57Bl/6J mice were food-deprived for 18 hrs. At the end of the fasting period, animals received either Rapatar (0.5 mg/kg) or PBS via gavage and 15 minutes later were allowed access to food. One hour later animals were sacrificed; livers were dissected, lysed in RIPA buffer and loaded on a 8% SDS-PAGE gel. After separation and transfer to a PVDF membrane, protein lysates were analyzed for mTOR activation by probing with an antibody against phospho-*p70 S6* Kinase (*Thr389*) (1:1000; Cell Signaling) and Actin (1:10000 Cell Signaling). After incubation with HRP conjugated secondary antibodies (Santa Cruz Biotechnologies), transferred proteins were visualized with the ECL detection kit (Jackson Research Laboratories).

### Histopathology

The mice were visually inspected for tumor development and weighed weekly. Animals showing deteriorating clinical status manifested by constant weight loss or visual tumor appearance were sacrificed and evaluated for gross pathological changes by complete necropsy. For histological evaluation, all tissues were fixed in 10% neutral formalin for 24 hours, and then transferred to 70% ethanol. Samples were embedded in paraffin, sectioned and stained with hema-toxylin and eosin. Histopathological examination was performed on tumors, gross lesions and target tissues using Zeiss AxioImager A1 with Axiocam MRc digital camera. The guidelines of Bethesda classification was used in determining the diagnosis [39].

### Statistical Analyses

Differences in survival and tumor incidence were evaluated by the Mantel-Cox log-rank test.
